# Frequency of RANTES gene polymorphisms and their association with incidence of malaria: a longitudinal study on children in Iganga district, Uganda

**DOI:** 10.1186/s12936-015-0875-0

**Published:** 2015-09-05

**Authors:** Catherine N. Lwanira, Mark Kaddu Mukasa, Göte Swedberg, Fred Kironde

**Affiliations:** School of Biomedical Sciences, College of Health Sciences, Makerere University, PO Box 7072, Kampala, Uganda; School of Medicine, College of Health Sciences, Makerere University, Kampala, Uganda; Department of Medical Biochemistry and Microbiology, Uppsala University, Uppsala, Sweden; Faculty of Health Sciences, Habib Medical School, Islamic University in Uganda (IUIU), Kampala Campus, Kampala, Uganda

**Keywords:** RANTES gene polymorphisms, *Plasmodium falciparum* malaria, Incidence

## Abstract

**Background:**

The severity and outcome of malaria is influenced by host immunity in which chemokines such as Regulated upon Activation, Normal T cell Expressed and Secreted (RANTES) play an important role. Previous studies show that variations in the RANTES gene affect RANTES protein production, hence altering host immunity. In this study, the relationship between presence of mutations in RANTES and incidence of malaria in a cohort of children living in a malaria-endemic area of Uganda was determined.

**Methods:**

This was a longitudinal study comprising of 423 children aged between 6 months and 9 years, who were actively followed up for 1 year. Malaria episodes occurring in the cohort children were detected and the affected children treated with national policy drug regimen. Mutations in the RANTES gene were determined by PCR–RFLP method and their frequencies were calculated. A multivariate negative binomial regression model was used to estimate the impact of RANTES mutations on malaria incidence. In all statistical tests, a P-value of <0.05 was considered as significant.

**Results:**

The frequencies of the −403A and In1.1C allele were 53.7 and 19.2 %, respectively. No mutations were found at the −28 locus. After adjustment of incidence rates for age, blood group, insecticide-treated bed net (ITN) use, malaria history and the sickle cell trait, 1n1.1T/C heterozygotes and homozygotes showed a non-significant trend towards higher incidence rates compared to wild-type individuals (IRR = 1.10; P = 0.55 and IRR = 1.25; P = 0.60, respectively). Similarly, there was no significant difference in malaria incidence rates between RANTES −403G/A heterozygotes or homozygotes and those without mutations (IRR = 1.09; P = 0.66 and IRR = 1.16; P = 0.50, respectively). No relation was seen between RANTES polymorphisms, baseline parasite densities and the time to first re-infection after administration of anti-malaria drugs.

**Conclusions:**

This study showed that the −403A mutation occurs in nearly half of the study population and the In1.1C allele occurs in one in every four children. Despite the high frequency of these mutations, there was no clear association with malaria incidence. Other studies evaluating more markers, that could potentially modulate RANTES gene transcription alongside other genetic modifiers of malaria susceptibility, may provide further explanations to these less dramatic findings.

## Background

*Plasmodium falciparum* malaria accounts for approximately 198 million clinical cases of malaria worldwide and 584,000 deaths annually [1]. A majority of these deaths occur in sub Saharan Africa, with over 78 % of all deaths happening in children under 5 years of age [1]. The development of naturally acquired immunity takes time and is associated with increasing age, which correlates with a reduction in mortality rates arising from severe forms of *P. falciparum* infection [2]. The development of this immunity still remains a mystery and as to why malaria episodes occur more frequently in some children compared to others raises further questions. Host genetic factors play an important role in reducing the risk to *Plasmodium* infection. The protective effect of the sickle cell trait (Hb AS) against both uncomplicated and severe malaria disease has been well documented [3–8]. It is, therefore, important to examine different host factors in order to further define their association with *P. falciparum* infection.

Regulatory cytokines and other mediators have also been reported to play a critical role in controlling parasitaemia and subsequent elimination of infection. Interferon-gamma (IFN γ), tumor necrosis factor (TNF), Interleukin 10 (IL-10), IL-17, IL-4, C–C chemokine RANTES (Regulated upon Activation, Normal T cell Expressed and Secreted), matrix metalloproteinases 8 (MMP8s) and tissue inhibitor of metalloproteinases 1 (TIMP1) have been linked to disease severity in malaria-infected individuals [9–16]. The mechanism of this immune modulation involves activation of leukocytes, recruitment of leukocytes and *Plasmodium*-infected erythrocytes causing occlusion of blood vessels, degradation of cell–cell junctions, blood–brain barrier dysfunction, interfering with formation of blood vessels and formation of blood cells or haematopoiesis [11, 12, 17–21]. Previous studies have shown that variations in the RANTES gene affect RANTES protein production and the subsequent host immune response towards a variety of infections [22, 23]. Low levels of the RANTES protein have also been observed in severe malaria due to acquisition of *Plasmodium* haemozoin by monocytes [24] or malaria-induced thrombocytopaenia [11] and have been associated with mortality among children with cerebral malaria [12].

The human RANTES gene is located on chromosome 17q11.2-q12, has a genomic size of 8.8 kb and encodes a protein of 8 kDa [22]. Among the several single nucleotide polymorphisms (SNPs) in the RANTES gene that have been reported before, the −403G/A and the −28C/G nucleotides located in the promoter region, along with the InI.1T/C present in the first intron, are the most polymorphic and appear to modify RANTES transcription [22]. The RANTES −28G variant was found to up-regulate RANTES expression in vitro [22, 23] and to be associated with delayed disease progression among HIV-infected adults [23, 25–27]. The In1.1C variant, which also occurs in strong linkage disequilibrium with −403 A allele, was associated with reduced RANTES transcription activity in vitro [22] and increased rate of AIDS progression [22, 28, 29], but was found to be protective against disease progression among Ugandan HIV adults [30]. The prevalence of these RANTES polymorphisms varies in different populations. The −403 A allele occurs predominantly among African populations [22, 28, 30], while the −28G allele is found to be more prevalent among Japanese [31] and Han Chinese populations [23], but scarcely distributed among Caucasians and African populations [22, 30]. RANTES levels were also found to vary by race with low values prevailing among African populations compared to Caucasians [32].

In several other studies, RANTES polymorphisms have been shown to affect the course of systemic lupus erythematosus [33], asthma [34], atopic dermatitis [35, 36], diabetic nephropathy [37], coronary artery disease [38], recurrent acute rejection [39], and sickle cell anaemia [40]. However, little is known of their role in malaria. Since these variants have been shown to modify RANTES protein expression and low levels of RANTES have been implicated in malaria, this study was designed to determine the relation between these variants and the incidence of malaria among children living in an endemic area. Accordingly, the aim of this study was (1) to determine the allelic and genotype prevalences of RANTES gene polymorphisms, namely −403G/A, −28C/G and In1.1T/C in a population of children from a malaria-endemic area (Iganga, Uganda); and, (2) to investigate the relationship between these polymorphisms with malaria incidence, parasite densities upon malaria diagnosis and the length of time to first re-infection following curative malaria treatment in this children’s cohort.

## Methods

### Study design and setting

This was a longitudinal study carried out in villages of Iganga district where children aged 6 months to 9 years were enrolled in order to determine malariometric indices during September 2008 and November 2009 towards preparation for the GMZ2 phase II malaria vaccine trial. From September 2008 to October 2008, a team of experienced home visitors approached households to systematically recruit children into the baseline study. Eligible children were enrolled into the study and followed up for a period of 1 year from November 2008 to November 2009. Iganga district is situated in south-eastern Uganda, about 25 km to the north of Lake Victoria and lies at an altitude of about 1138 m above sea level at latitude: 00°36′ North and longitude: 33°30′ East. Malaria transmission is this area is holo-endemic (intense and perennial), with transmission peaks seen following the major rains, which normally occur between April to June and September to December [41]. The annual entomological inoculation rate (EIR) is not known, but is estimated to be 562 infective bites per person per year in the neighbouring district of Tororo [42]. The study cohort was recruited from a community living within six villages of Iganga district that are in close proximity to the malaria study clinic located at Makerere University Iganga/Mayuge Demographic Surveillance Site (MaK-DSS). No interventional studies were undertaken in this study area at the time this study cohort was assembled. Inclusion criteria of cohort study were as follows; (1) age 6 months to 9 years; (2) agreement to come to study clinic for any febrile episode or illness; (3) agreement to avoid medications administered outside the study; (4) agreement to remain in study area during the twelve months follow up; (5) absence of known chronic disease and (6) written informed consent provided by parent or guardian. Severely malnourished children (below −3z scores of the median WHO growth standards) [43] were excluded. Follow-up started when children fulfilled all of the selection criteria and were free of symptomatic malaria. The villages were divided by convenience into active (nearby) villages and passive (more remote) villages.

### Clinical assessment and determination of malaria incidence

A baseline survey was carried out and eligible children were enrolled into this cohort. At the time of enrolment, a physical examination was performed by study physicians. Baseline demographic data were collected from guardians through a questionnaire-based interview, including malariometric indices. After the baseline survey, parents or guardians were instructed to bring their children to the malaria clinic based at Iganga Hospital whenever they felt unwell so as to avoid using any drugs not administered or approved by a study physician. A team of field workers visited the enrolled children at home every fortnight. Those children found unwell were referred to the clinic for care or referral in case of severe illness. Malaria was defined as any *P. falciparum* parasitaemia plus a tympanic temperature of ≥38.0 °C or a history of fever (within the past 48 h). Thick and thin peripheral blood slides were stained with Giemsa and examined under a microscope. Parasite densities were calculated by counting the number of asexual parasites per 200 white blood cells (WBC) and assuming a WBC count of 8000/µL blood [44]. In addition, approximately 2 ml of blood sample were drawn and mixed with ethylenediaminetetracetic acid (EDTA) anticoagulant for subsequent analysis of DNA. Severe malaria was defined as having hyper parasitaemia (>5 % parasitized erythrocytes or >250,000 parasites/µL) and any of the danger signs including a core body temperature >40 °C, severe anaemia (haemoglobin <5 g/dl),hyper bilirubinemia (total bilirubin >2.5 mg/dl), prostration or weakness, impaired consciousness, respiratory distress, hypoglycaemia (blood sugar <40 mg/dL), acidosis, cerebral malaria or other additional sign as specified in definition for severe malaria [45, 46]. Children with confirmed malaria were treated with artemether-lumefantrine (Coartem^®^) according to the national treatment guidelines and those with severe malaria were admitted for care at Iganga Hospital acute care unit. Time at risk for new infection was considered as the duration of study participation excluding 14 days after each episode of malaria.

### Genomic DNA isolation

Genomic DNA was extracted from blood leukocytes using E.Z.N.A Blood DNA kit as outlined by the manufacturer’s protocol (Omega Bio-tek, USA). Approximately 0.1 µg of genomic DNA was used for the genotyping assays.

### Detection of RANTES gene polymorphisms

Polymorphisms were detected using polymerase chain reaction (PCR) amplification followed by restriction endonuclease digestion (RFLP) as described elsewhere [23, 47, 48]. For each reaction, 1–2 µL of the extracted DNA sample was incubated with DreamTaq DNA polymerase (Thermo scientific Inc, USA), 0.5 µM of each primer, 100 µM dNTPs and 2.0 mM MgCl_2_. After first-round PCR, about 5 µL of each reaction was digested with restriction endonucleases and products were subjected to electrophoresis on 2.5 % agarose gels (Thermo scientific Inc, USA) and visualized with ethidium bromide. Genotypes were assessed by comparing the sizes of reaction products and controls after digestion.

### Sickle cell genotyping

Sickle cell genotyping was based on characterization of haemoglobin AS (Hb AS) using nested PCR followed by RFLP as previously described by Parikh et al. [49]. In the first round, approximately 1–2 µL of the extracted DNA was incubated with DreamTaq DNA polymerase (Thermo scientific Inc, USA), 0.1 µM of each primer, 200 µM dNTPs and 2.0 mM MgCl_2_.PCR products from the first round were subjected to a second amplification using DreamTaq DNA polymerase (Thermo scientific Inc, USA), 0.05 µM of each primer, 200 µM dNTPs and 2.0 mM MgCl_2_. After second-round PCR, products were digested with restriction endonucleases and subjected to electrophoresis on 2.5 % agarose gels (Thermo scientific Inc, USA) containing ethidium bromide. As described above, genotypes were assessed by comparing the sizes of reaction products and controls after digestion.

### Data management and analysis

Data were cleaned, coded and entered into Microsoft Office Access ™ 2007. Descriptive statistics, Chi square tests and multivariate analysis were carried out using Stata12.0 (Stata Corp, College Station, TX, USA). Allele and genotype frequencies were calculated in accordance with the Hardy–Weinberg principle [50]. The association between RANTES genotype and malaria incidence was estimated using a multivariate negative binomial regression model. Initially, a bivariate analysis was performed to determine the relationship between each demographic/clinical characteristic and malaria incidence. The final multivariate analysis model included the RANTES genotype and all identified predictors of malaria incidence (age, malaria history, ITN use and the sickle cell trait). Adjusted incidence rates ratios (IRRs), P values and 95 % confidence intervals were calculated. The Wilcoxon rank sum test was used to compare the distribution of parasite densities across the different RANTES genotypes.

The effect of RANTES polymorphisms on the length of time to first re-infection following administration of anti-malarial drug was estimated using Kaplan–Meier non-parametric survival analysis. The log rank test was used to evaluate differences in the survival times between the different RANTES genotypes. Cox proportion hazard regression model was used to control for other independent predictors of length of time to re infection (age, malaria history and ITN use) and adjusted hazard ratios (aHRs) were calculated. For all statistical tests, two-sided p values of less than 0.05 were assumed to show statistical significance.

### Ethical considerations

The clinical study and all study protocols were approved by the School of Medicine Research and Ethics Committee of the College of Health Sciences, Makerere University and by the Uganda National Council for Science and Technology (approval number HS 765). All participants provided written informed consent. All children received appropriate treatment for other attendant medical conditions.

## Results

### Study population

**Fig. 1:**
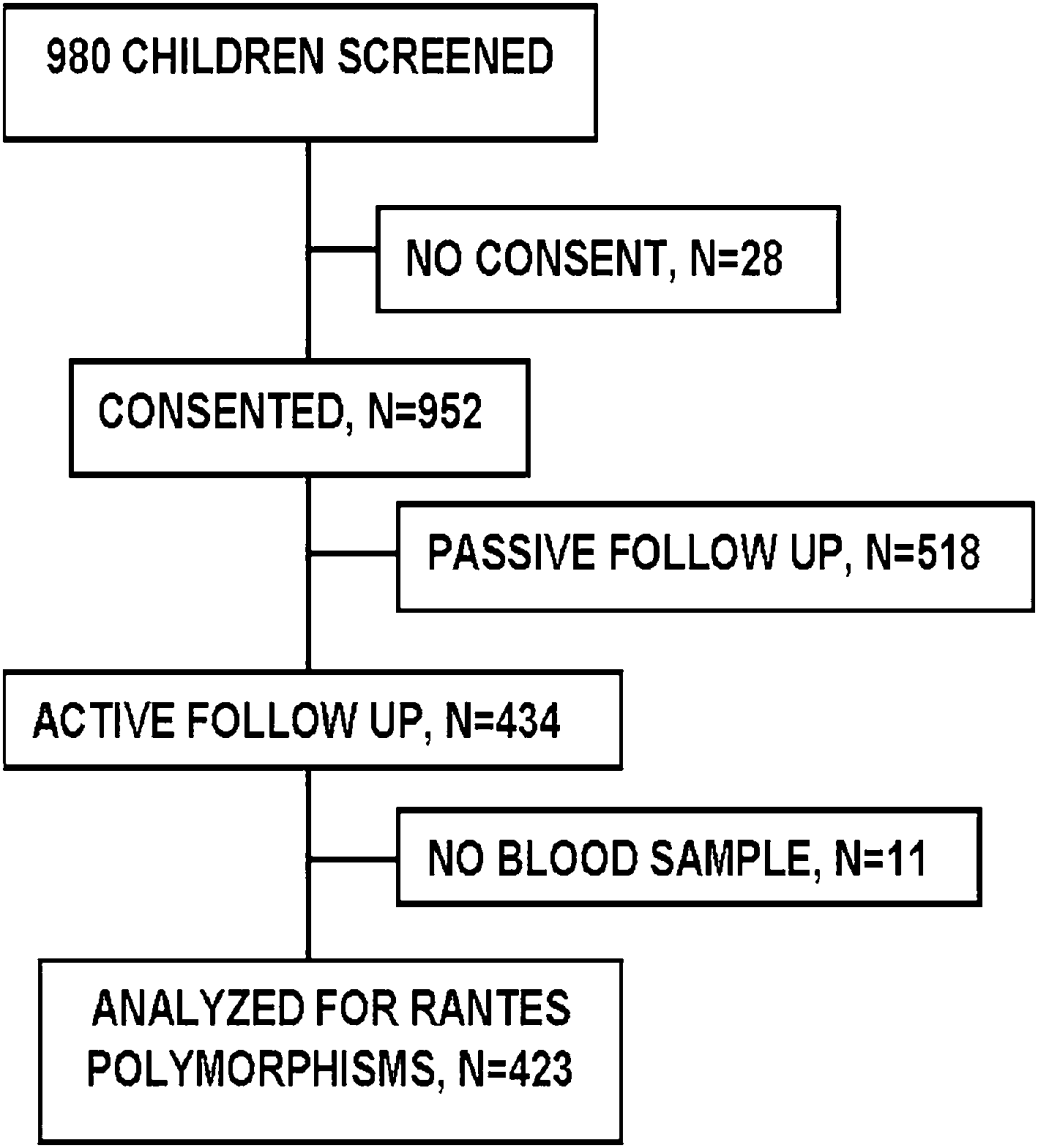
Participant flow chart. Figure showing the 423 children recruited and actively followed-up who provided samples for RANTES gene polymorphisms analysis

**Fig. 2:**
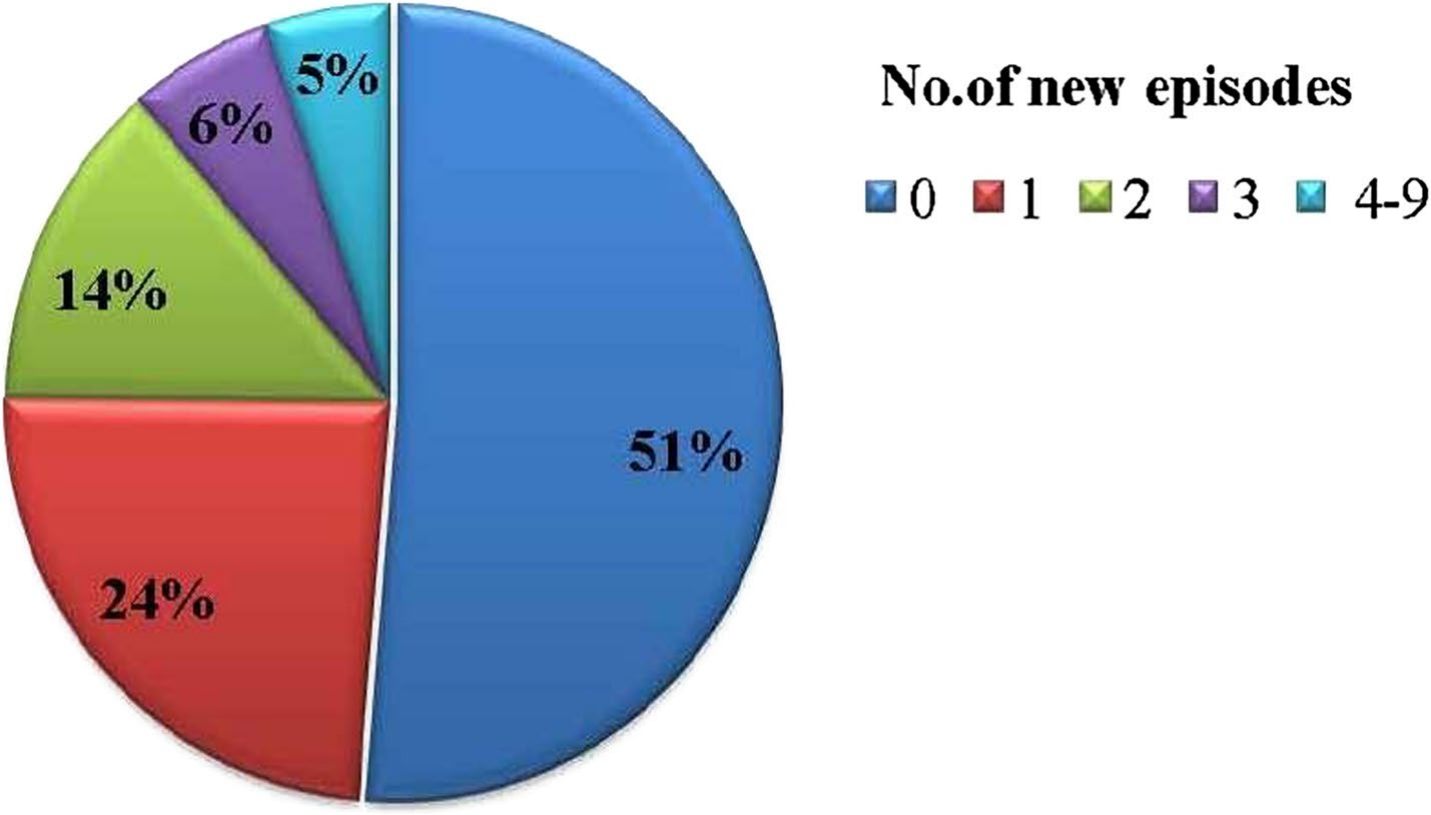
Incidence of malaria among the study participants. 51 % of the study participants had no malaria episodes during the longitudinal follow-up. 5 % experienced 4–9 malaria episodes during the follow-up

A total of 980 children were screened for baseline studies to determine malariometric indices at Makerere University Iganga-Mayuge Demographic study site, Iganga Hospital during September 2008 and November 2009 (Fig. 1) towards preparation for the GMZ2 phase II malaria vaccine trial (Fig. 1). Twenty-eight children were excluded because of guardian unwillingness to participate in the follow-up study. A sample of 434 for each arm was estimated based on assumptions of malaria incidence of 15 % in the active arm, with 95 % confidence interval, and ability to detect a vaccine efficacy of 30 %. However, assuming 20 % loss to follow up in the passive arm, a total sample of 518 was considered. Accordingly, of the remaining 952 children, 518 (54.4 %) were monitored passively and 434 (45.6 %) were followed up by active surveillance for 1 year and the incidence of malaria per child was determined. Eleven of the 434 children (2.5 %) would not provide an adequate blood sample for subsequent analysis of DNA. In order to determine the association between RANTES polymorphisms and malaria incidence, only 423 children who had been actively followed-up were included in this study. The majority of study participants (96.7 %) were of Basoga ethnic background, while the rest constituted other tribes. Baseline characteristics were as follows: mean age, 3.9 years (SD ± 2.3); sex, 52.7 % male and 47.3 % female; mean haemoglobin, 12 g/dL (SD ± 1.5); the predominant blood groups, O+ (39.4 %) and B+ (30.4 %); 26.6 % carried the sickle cell trait. A majority of the study participants (94.6 %) reported to have had history of malaria in the 6 months prior to study enrolment, 88.2 % reported to be using an insecticide-treated bed net (ITN) and 95.3 % reported having administered an anti-malarial drug previously (Table 1). No malaria episodes were registered among 217 out of 423 children (51.3 %) after a year of longitudinal follow-up. Among those with episodes (206 children), the range was one to nine episodes per child with the distribution as shown in Fig. 2.

**Table 1:** Demographic and clinical characteristics of the study population

Age in years (mean, SD)	3.92 ± 2.32
Sex, n (%)	
Male	223 (52.7)
Female	200 (47.3)
Weight in kg (mean, SD)	15.47 ± 5.21
Blood group, n (%)	
O+	166 (39.4)
A+	96 (22.8)
B+	128 (30.4)
AB	27 (6.4)
Non O (A+ B+ AB + others)	255 (60.6)
Sickle cell genotype, n (%)	
Wild type (Hb AA)	303 (73.2)
Heterozygous (Hb AS)	110 (26.6)
Homozygous (Hb SS)	1 (0.2)
Mean haemoglobin, SD (g/dL)	11.97 ± 1.46
Malaria history, n (%)	
Yes	400 (94.6)
No	15 (3.5)
Not sure	8 (1.9)
Reported ITN use, n (%)	
Yes	373 (88.2)
No	50 (11.8)
Reported previous anti-malarial drug use, n (%)	
Yes	403 (95.3)
No	14 (3.3)
Not sure	6 (1.4)

### Distribution of RANTES polymorphisms

PCR for genotyping of the three RANTES polymorphic sites was performed for all individuals. The success rate was 96 %. Out of the 406 children who were successfully genotyped for the RANTES −403 G/A polymorphism, 224 (55.2 %) had the RANTES GA genotype, 26.1 % carried the AA genotype while the remaining (18.7 %) had the wild type genotype. For the In1.1T/C polymorphisms, only 3.7 % carried homozygous mutations (CC), 30.9 % carried the heterozygous (TC) genotype and a majority (65.4 %) had the wild-type (TT) genotype. The overall prevalence of the respective markers is shown in Table 2. No polymorphisms at all were seen for the −28 C/G marker. The calculated allele frequencies for −403 G/A and In1.1 T/C polymorphisms were in agreement with earlier reports [22] and the distributions of the genotypes were close to the Hardy–Weinberg equilibrium. The two markers (In1.1 T/C and −403 G/A) showed a clear linkage disequilibrium, all cases with In1.1 mutations also showed −403 mutations (Table 3).

**Table 2:** Genotype and allele frequencies of the RANTES −403G/A, RANTES −28C/G and In1.1T/C polymorphisms

RANTES 403G/A (n = 406)		RANTES −28C/G (n = 403)		RANTES In1.1T/C (n = 404)	
Genotype distribution, n (%)					
GG	76 (18.7)	CC	403	TT	264 (65.4)
GA	224 (55.2)	CG	0	TC	125 (30.9)
AA	106 (26.1)	GG	0	CC	15 (3.7)
Allele frequency					
G	0.46	C	1.00	T	0.81
A	0.54	G	0.00	C	0.19

**Table 3:** Distribution of RANTES In1.1/403 compound genotypes among the study population

RANTES In1.1/403 genotype	Frequency	Percentage (%)
wt/wt	75	17.8
het/het	79	18.7
het/hom	42	9.9
hom/het	1	0.2
hom/hom	14	3.3
wt/^a^ any	212	50.1

*Wt* wild type, *het* heterozygous, *hom* homozygous

^a^Any-homozygous or heterozygous

### Effect of RANTES polymorphisms on malaria incidence and baseline parasite densities

A single across groups test (ANOVA) was initially performed to determine differences in the distribution of malaria incidence across the RANTES genotypes. Crude malaria incidence rates/person/year were 0.91 for In1.1T/C wild-type, 0.94 for heterozygous and 0.73 for homozygous individuals. There was no significant difference in the distribution of malaria incidence across the three genotypes (P = 0.76). Since there are known predictors of malaria incidence that needed to be adjusted for a multivariate negative binomial regression analysis was done in order to evaluate the association between RANTES polymorphisms and malaria incidence. An initial bivariate (unadjusted) analysis identified age, malaria history, ITN use and sickle cell trait (Table 4) as independent predictors of malaria incidence. After adjusting for the named confounders, the incidence rate for 1n1.1T/C heterozygotes was 1.1 times the incidence rate for wild-type individuals (P = 0.55) and that of homozygotes was 1.25 times the incidence rate for those without mutations (P = 0.6). Median parasite densities were as follows; wild type, 775 [inter quartile range (IQR) 225–2525] parasites/µL; heterozygote, 373 (IQR 150–2250) parasites/µL; homozygote, 2450 (IQR 925–11,000) parasites/µL. There was no difference in the median parasite densities between 1n1.1T/C heterozygotes and wild types (P = 0.26) or homozygotes and wild type individuals (P = 0.17).

**Table 4:** Malaria incidence and other demographic characteristics

Variable	Malaria		Number new episodes	Crude incidence rate ratio	P value	95 % CI
	No	Yes				
Age						
0.5–1 years	31	37	65	Reference	–	–
>1–5 years	121	123	272	0.85	0.397	0.590–1.233
>5–9 years	64	46	77	0.57	0.013*	0.371–0.887
Blood group						
A+	56	41	77	Reference	–	–
B+	73	57	109	1.06	0.768	0.713–1.581
O+	78	87	184	1.38	0.086	0.955–2.000
AB+	10	17	36	1.66	0.091	0.922–2.997
O−	1	1	4	2.49	0.306	0.433–14.340
B−	0	1	1	1.24	0.878	0.074–20.906
AB−	0	1	1	1.24	0.878	0.074–20.906
Sickle cell genotype						
Wild type	158	145	289	Reference		
Heterozygous	64	46	110	0.78	0.394	0.755–1.427
Malaria history	201	199	412	7.33	0.008*	1.674–32.104
Reported anti-malarial drug use	206	197	386	1.68	0.247	0.698–4.043
Reported ITN use	184	189	377	2.69	0.001*	1.522–4.752

For the RANTES −403G/A polymorphism, crude annual malaria incidence rates were 0.83 for wild-type, 1.07 for heterozygotes and 0.97 for homozygous individuals. There was no difference in the distribution of malaria incidence across the RANTES −403G/A genotypes (P = 0.47). The adjusted incident rate for RANTES −403G/A heterozygotes was 1.09 times the incidence rate for wild-type individuals (P = 0.66). Similarly, homozygotes showed a non-significant trend towards higher incidence rates than those carrying the wild type genotype (IRR = 1.16; P = 0.50) as shown in Table 5. Median parasite densities were as follows; wild type, 488 (IQR 213–2188) parasites/µL; heterozygote, 563 (IQR 200–2800) parasites/µL; homozygote, 825 (IQR 250–2450) parasites/µL. There was no difference in the geometric mean parasite densities between −403G/A heterozygotes and wild types (P = 0.98) or between homozygotes and wild type individuals (P = 0.82). Since the two markers (−403G/A and In1.1T/C) demonstrated a clear linkage disequilibrium, another multivariate analysis was done with linking markers. There was no statistically significant association between any combination of mutations with malaria incidence (Table 6).

**Table 5:** Effect of RANTES polymorphisms on incidence of malaria

RANTES genotype	Malaria		Number new episodes	Adjusted incidence rate ratio	*P*	95 % CI	Median parasite density^a^	*P*
	No	Yes						
RANTES 403G/A								
GG	42	34	63	Reference	–	–	488	Reference
GA	108	117	240	1.09	0.664	0.744–1.590	563	0.981
AA	57	49	103	1.16	0.500	0.753–1.788	825	0.815
RANTES In1.1T/C								
TT	143	122	240	Reference	–	–	775	Reference
TC	63	62	118	1.10	0.344	0.804–1.506	373	0.258
CC	9	6	11	1.25	0.560	0.541–2.888	2450	0.166

^a^Median parasite density (parasites/µL) at the time of diagnosis of symptomatic malaria

**Table 6:** RANTES In1.1/403 compound genotypes and malaria incidence

RANTES In1.1/403 genotype	Malaria		No. of new episodes	Adjusted incident rate ratio	P value	95 % CI
	No	Yes				
wt/wt	42	33	61	Reference	–	–
het/het	36	43	89	0.731	0.162	0.472–1.133
het/hom	25	18	28	1.171	0.609	0.640–2.142
hom/hom	8	6	11	0.935	0.879	0.394–2.220

### Effect of RANTES polymorphisms on time to first re-infection

**Fig. 3:**
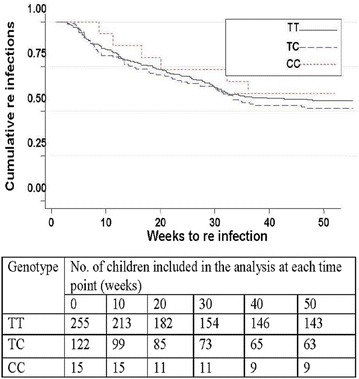
Kaplan–Meier plots for re infection by RANTES INT1.1 genotype (TT, TC or CC). Cumulative re-infections are plotted against weeks to the next infection. The study participants were actively followed-up with visits once every 2 weeks at their homes, to obtain information about re-infection. Using a Cox proportion hazard regression model, adjusting for age, malaria history and ITN use, the predictors of length of time to first re-infection for TT (wild- type); TC (heterozygous) and CC (homozygous) were not statistically different with p-value of 0.28 and 0.67, respectively

The impact of RANTES polymorphisms upon time to first re-infection after administration of malaria treatment was also evaluated. The average time (weeks) to first re-infection was 13.4 for RANTES In1.1T/C wild-type individuals, 12.7 for heterozygotes and 16.7 for homozygotes. There was no significant difference in the length of time to first re-infection between heterozygote and wild-type genotypes (P = 0.74) or between homozygote and wild-type genotypes (P = 0.34). After adjusting HRs for age, malaria history and ITN use, the hazard for re-infection did not differ significantly between heterozygotes and wild-type genotypes (HR = 1.19; P = 0.28) or between the In1.1C homozygotes and wildtypes (HR = 1.2; P = 0.67) (Fig. 3).

**Fig. 4:**
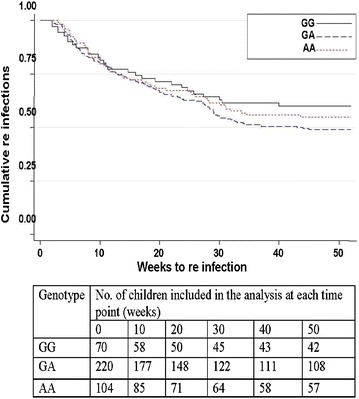
Kaplan–Meier plots for re-infection by RANTES −403 genotype (GG, GA or AA). Cumulative re-infections are plotted against weeks to the next infection. Following an active follow-up with home visits once every 2 weeks, information about re-infection was obtained from the study participants. Using a Cox proportion hazard regression model, adjusting for age, malaria history and ITN use, the predictors of length of time to first re-infection for GG (wild-type); GA (heterozygous) and AA (homozygous) were not statistically different with p-value of 0.21 and 0.42, respectively

The average time (weeks) to first re-infection was 10.8 for RANTES −403 G/A wild-type individuals, 13.4 for heterozygotes and 11.4 for homozygotes. The was no difference in the length of time to first re-infection between heterozygotes and wild-type genotypes (P = 0.29) or between homozygotes and wild-type individuals (P = 0.44). Additionally, no significant difference was seen in the hazard for re-infection for wild types vs heterozygotes (HR = 1.31; P = 0.21) or between the wildtypes and −403A homozygotes (HR = 1.22; P = 0.42) (Fig. 4).

## Discussion

In this study, the relationship between the presence of mutations in RANTES and incidence of malaria in a cohort of children living in a malaria-endemic area of Uganda was determined. Three SNPs (−28C/G, −403G/A and In1.1T/C) in the RANTES gene region were examined. These SNPs have been shown to modulate RANTES gene transcription in vitro [22] and often demonstrated as important determinants of disease susceptibility and clinical outcomes for a variety of infections [22, 33–35, 37–40]. Yet, no relation with incidence of malaria has been reported before. Of the three previously described polymorphic sites, mutations were present at only two sites, the −403G/A and the In1.1T/C markers. The frequencies of the −403 A and In1.1C alleles were 53.7 and 19.2 %, respectively. These rates are close to the frequencies of 43 and 20 % for −403 A and In1.1C, respectively, reported elsewhere among a population of African Americans [22] and comparable to those found in Uganda [30] even though these studies were conducted among populations from different geographical settings. Also, notably, the results showed that the two markers occur in strong linkage disequilibrium; all cases with In1.1 mutations also showed −403 mutations. This finding is consistent with other reports that examined other populations in different study settings [22]. The rare −28G allele is found predominantly among Japanese [31] and Han Chinese populations [23], but scarcely distributed among Caucasian, African American [22] and Ugandan populations [30]. In this study, no mutations were found at the −28C loci consistent with studies conducted elsewhere [22].

The In1.1C allele, which occurs in strong linkage disequilibrium with the −403A allele, has been shown to lead to down-regulation of RANTES and lower levels of the cytokine [22, 23]. Low levels of RANTES have previously been associated with severe malaria [11, 12], but no direct relation with any incidence of malaria was seen before. In the present study, none of the RANTES mutations investigated showed any significant effect on malaria incidence, baseline parasite densities or time to first re-infection. In that context, the high frequency of the −403 mutation is surprising, since, as with many other polymorphisms such as sickle cell trait, malaria is cited as a factor that selects for a disease-related gene variant [5, 6]. Of course, RANTES is important in many other infections as well, but it is still hard to explain that lower levels of a chemokine could possibly be protective against common infections. The third marker, at −28, was found to be wild type in all children included in the present study. Absence of the −28 mutation, is a little surprising. This mutation up-regulates expression [22, 23], and would confer better protection. However, higher levels of RANTES may be detrimental for other reasons [32]. This is also in line with high prevalence of the −403 mutation, which reduces RANTES levels. Other studies confirm the higher incidence of the −403 mutation in African populations [22, 28, 30] and could indicate a selective force for low levels of RANTES. This is also consistent with other reports in which lower levels of RANTES have been observed in African populations compared to other populations [32]. Given the role of RANTES in protection against infectious diseases, these findings remain hard to explain.

In other disease conditions such as HIV where RANTES polymorphisms have been studied, there are still contrasting reports on the influence of these variants on susceptibility and disease progression. The strong down-regulatory activity on RANTES transcription that is afforded by −403 and In1.1 mutations has been shown to lead to increased susceptibility to HIV infection [29] and increased rate of disease progression [22, 48]. In other studies, no associations between these variants and HIV infection could be observed [28] and elsewhere, In1.1C homozygosity was associated with delayed disease progression among adult HIV-positive patients [30]. These findings suggest that the role of these variants in determining disease susceptibility and clinical outcomes in different study settings is still not clearly understood. Other markers within the RANTES gene that could potentially modulate RANTES expression may need to be evaluated in order to understand the observable differences in epidemiological patterns. This may provide an insight into a clearer understanding of the role of RANTES polymorphisms in other diseases like malaria.

In the present study, previous history of malaria prior to recruitment was recorded as well. Most children reported previous experience of malaria, and these showed a higher incidence rate than those who were earlier free from the disease. These findings may suggest a genetic basis for susceptibility to malaria, which of course is most likely multifactorial, and dependent on other markers besides RANTES. A small number of children had many episodes of malaria during the study, up to nine during 1 year. At each incidence, the children were treated and cured from malaria, but they obviously get re-infected rapidly, and obviously do not develop protective immunity. At the same time, many children stayed free from malaria for the whole year, and the majority of those with malaria had only one episode. These children live under very similar conditions, so they should be exposed to infectious mosquito bites at similar rates. In this study, there was no significant relation between the RANTES polymorphisms and malaria incidence. Given that malaria susceptibility involves a complex interplay of other genetic modifiers, such as sickle cell trait, G6PD deficiency, among others [5, 6], the impact of RANTES polymorphisms alone on malaria incidence may not be evident. Sickle cell heterozygotes showed lower incidence of malaria compared to those with normal haemoglobin. The O+ blood group type known for protection against severe *P. falciparum* malaria [51, 52] instead showed a trend towards higher incidence of malaria, a variation that may relate to differences in the impact of the O+ blood group type upon severe malaria rather than the actual susceptibility to malaria. Controlling for these factors still yielded no significant associations between the RANTES polymorphisms and malaria incidence. Thus, additional studies evaluating other markers that could potentially modify RANTES gene expression alongside other biological interactions between other genetic modifiers and their impact on malaria susceptibility may provide a clearer understanding of these less dramatic associations.

There were some limitations to this study. Information on known preventive measures and prior anti-malarial drug use was obtained by questionnaire-based interviews, which could have influenced the outcome of the multivariate analysis since known protective measures unexpectedly scored as risk factors of malaria. Additionally, this study looked at a single gene (RANTES). Additional modifying genetic factors other than the sickle cell trait were not adjusted for.

## Conclusions

Results of this study show that the −403A mutation occurs in nearly half the study population and In1.1C allele occurs in one in every four children. Despite the high frequency of these mutations, there was no clear association with malaria incidence. Since both polymorphisms lead to down-regulation of RANTES expression and expected lower levels of RANTES, these findings remain hard to explain. There were no mutations at the −28 marker. This marker has previously been shown to lead to increased RANTES expression. Thus, absence of −28 mutation in the Uganda study population is quite surprising. Evaluating other markers that could potentially modulate RANTES gene transcription may provide further explanations to these less dramatic findings. Additionally, since there are several other markers that have been implicated in pathogenesis of malaria, the impact of RANTES polymorphisms alone on malaria incidence may be difficult to evaluate. Therefore, other larger studies looking at the interplay between other genetic modifiers and malaria susceptibility may provide clearer understanding of these findings.

## Abbreviations

IFN-γ: interferon-gamma
TNF: tumor necrosis factor
IL-10: interleukin-10
IL-17: interleukin-17
IL-4: interleukin-4
RANTES: regulated upon activation, normal T cell expressed and secreted
MMP8 s: matrix metalloproteinases 8
TIMP1: tissue inhibitor of metalloproteinases 1
SNP: single nucleotide polymorphism
HIV: human immunodeficiency virus
AIDS: acquired immuno deficiency syndrome
WHO: World Health Organization
EIR: entomological innoculation rate
WBC: white blood cells
EDTA: ethylene diamine tetraacetic acid
DNA: deoxyribonucleic acid
PCR: polymerase chain reaction
RFLP: restriction fragment length polymorphism
IRR: incident rate ratio
IQR: inter quartile range
HR: hazard ratio
ITN: insecticide treated bed net
ANOVA: analysis of variance
